# *catena*-Poly[[[[3-(2-pyrid­yl)-1*H*-pyrazole]manganese(II)]-μ-oxalato] sesquihydrate]

**DOI:** 10.1107/S1600536809044298

**Published:** 2009-10-31

**Authors:** Zhe An, Ling Zhu

**Affiliations:** a School of Chemistry and Life Science, Maoming University, Maoming 525000, People’s Republic of China

## Abstract

In the title compound, {[Mn(C_2_O_4_)(C_8_H_7_N_3_)]·1.5H_2_O}_*n*_, the Mn^II^ ion is chelated by two *O*,*O*′-bidentate oxalate ions and an *N*,*N*′-bidentate 3-(2-pyrid­yl)pyrazole mol­ecule, resulting in a distorted *cis*-MnN_2_O_4_ octa­hedral geometry for the metal ion. The bridging oxalate ions generate wave-like polymeric chains propagating in [001]. The packing is consolidated by N—H⋯O and O—H⋯O hydrogen bonds. One of the water O atoms lies on a crystallographic twofold axis.

## Related literature

For coordination compounds with pyridyl-pyrazolide ligands, see: Ward *et al.* (1998 ▶, 2001 ▶).
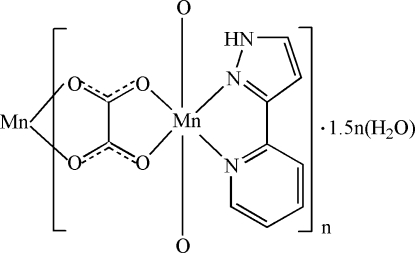


## Experimental

### 

#### Crystal data

[Mn(C_2_O_4_)(C_8_H_7_N_3_)]·1.5H_2_O
*M* *_r_* = 315.15
Monoclinic, 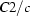

*a* = 29.460 (8) Å
*b* = 9.236 (3) Å
*c* = 9.875 (3) Å
β = 102.706 (5)°
*V* = 2621.0 (13) Å^3^
*Z* = 8
Mo *K*α radiation
μ = 1.03 mm^−1^
*T* = 296 K
0.43 × 0.28 × 0.22 mm

#### Data collection

Bruker SMART APEX CCD diffractometer
Absorption correction: multi-scan (*SADABS*; Bruker, 2005 ▶) *T*_min_ = 0.665, *T*_max_ = 0.805
6809 measured reflections
2438 independent reflections
2004 reflections with *I* > 2σ(*I*)
*R*_int_ = 0.020

#### Refinement

*R*[*F* ^2^ > 2σ(*F* ^2^)] = 0.027
*wR*(*F* ^2^) = 0.078
*S* = 1.00
2438 reflections
186 parameters
H atoms treated by a mixture of independent and constrained refinement
Δρ_max_ = 0.22 e Å^−3^
Δρ_min_ = −0.17 e Å^−3^

### 

Data collection: *SMART* or APEX2? (Bruker, 2005 ▶); cell refinement: *SAINT* (Bruker, 2005 ▶); data reduction: *SAINT*; program(s) used to solve structure: *SHELXS97* (Sheldrick, 2008 ▶); program(s) used to refine structure: *SHELXL97* (Sheldrick, 2008 ▶); molecular graphics: *SHELXTL* (Sheldrick, 2008 ▶); software used to prepare material for publication: *SHELXL97*.

## Supplementary Material

Crystal structure: contains datablocks I, global. DOI: 10.1107/S1600536809044298/hb5162sup1.cif

Structure factors: contains datablocks I. DOI: 10.1107/S1600536809044298/hb5162Isup2.hkl

Additional supplementary materials:  crystallographic information; 3D view; checkCIF report

## Figures and Tables

**Table:** 

Mn1—N1	2.280 (4)
Mn1—N2	2.223 (4)
Mn1—O4^i^	2.150 (3)
Mn1—O2	2.168 (3)
Mn1—O1	2.191 (4)
Mn1—O3^i^	2.208 (3)

**Table:** 

N2—Mn1—N1	73.01 (16)

Symmetry code: (i) 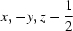.

**Table 2:** Hydrogen-bond geometry (Å, °)

*D*—H⋯*A*	*D*—H	H⋯*A*	*D*⋯*A*	*D*—H⋯*A*
N3—H3*A*⋯O1*W*	0.86	1.89	2.748 (7)	175
O1*W*—H1*W*⋯O1^ii^	0.83 (5)	2.08 (4)	2.851 (6)	155 (6)
O1*W*—H2*W*⋯O2*W*^iii^	0.82 (4)	2.10 (5)	2.819 (6)	148 (6)
O2*W*—H3*W*⋯O3^iv^	0.82 (5)	2.06 (4)	2.823 (4)	156 (6)

Symmetry codes: (ii) 
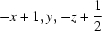
; (iii) 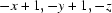; (iv) 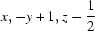.

**Fig. 1.**
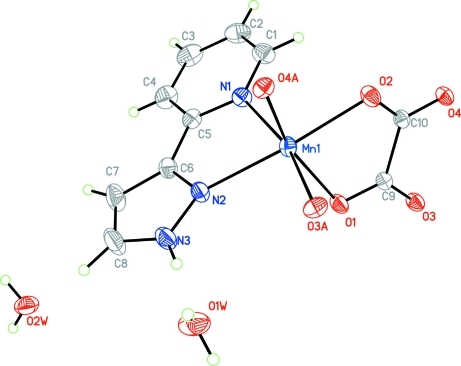
A view of (I) with the unique-atom labelling scheme. Displacement ellipsoids are drawn at the 30% probability level.

**Fig. 2.**
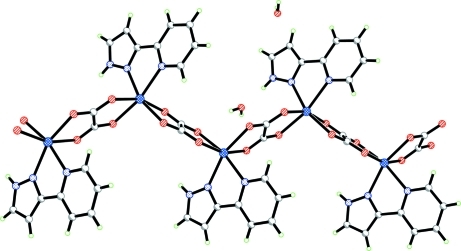
A view of (I) showing the extended chain strcuture.

**Fig. 3.**
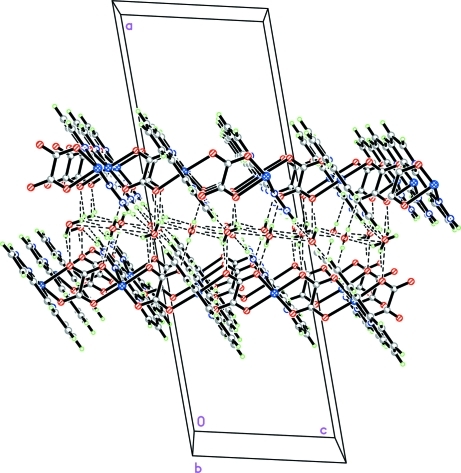
A view of (I) showing the packing.

## supplementary crystallographic information

### Comment

The tridentate ligand 3-(2-pyridyl)pyrazole and its derivatives have been used widely in the construction of supramolecular architectures by way of metal-organic coordination (Ward, Fleming *et al.* 1998; Ward, 2001).

As a continuation of these studies, we now report the crystal structure of the title complex, (I).

The Mn ion is hexcoordianted, chelated by two oxalate and one 3-(2-pyridyl)pyrazole ligand (Table 1). While each oxalate ligand acts as one bridige to chalate two Mn ions, forming one wave-like line with Mn···Mn distance being 5.652 /%A, shown in Figure 2. The structure is consolidated by N—H···O and O—H···O hydrogen bonds (Table 2, Figure 3).

### Experimental

A mixture of Mn(CH_3_COO)_2_.4H_2_O (1 mmol), 3-(2-pyridyl)pyrazole (1 mmol), oxalic acid (1 mmol), sodium hydroxide (1 mmol) and H_2_O (10 ml) was stirred for 30 min in air. The mixture was then transferred to a 25 ml Teflon-lined hydrothermal bomb. The bomb was kept at 433 K for 72 h under autogenous pressure. Upon cooling, pink prisms of (I) were obtained from the reaction mixture.

### Refinement

The C-bound H atoms were geometrically planced (C—H = 0.93/%A) and refined as riding with *U*_iso_ = 1.2Ueq(C). The N– and O-bound H atoms were located in difference maps and refined with distance restraints: N—H = 0.97 (1)/%A, O—H = 0.82 (2)/%A, H···H = 1.38 (2)/%A.

### Crystal data

**Table:**

[Mn(C_2_O_4_)(C_8_H_7_N_3_)]·1.5H_2_O	*F*(000) = 1280
*M**_r_* = 315.15	*D*_x_ = 1.597 Mg m^−^^3^
Monoclinic, *C*2/*c*	Mo *K*α radiation, λ = 0.71073 Å
Hall symbol: -C 2yc	Cell parameters from 2634 reflections
*a* = 29.460 (8) Å	θ = 2.8–25.4°
*b* = 9.236 (3) Å	µ = 1.03 mm^−^^1^
*c* = 9.875 (3) Å	*T* = 296 K
β = 102.706 (5)°	Block, pink
*V* = 2621.0 (13) Å^3^	0.43 × 0.28 × 0.22 mm
*Z* = 8	

### Data collection

**Table:**

Bruker APEXII CCD diffractometer	2438 independent reflections
Radiation source: fine-focus sealed tube	2004 reflections with *I* > 2σ(*I*)
graphite	*R*_int_ = 0.020
φ and ω scans	θ_max_ = 25.5°, θ_min_ = 2.3°
Absorption correction: multi-scan (*SADABS*; Bruker, 2005)	*h* = −35→35
*T*_min_ = 0.665, *T*_max_ = 0.805	*k* = −10→11
6809 measured reflections	*l* = −9→11

### Refinement

**Table:**

Refinement on *F*^2^	Primary atom site location: structure-invariant direct methods
Least-squares matrix: full	Secondary atom site location: difference Fourier map
*R*[*F*^2^ > 2σ(*F*^2^)] = 0.027	Hydrogen site location: inferred from neighbouring sites
*wR*(*F*^2^) = 0.078	H atoms treated by a mixture of independent and constrained refinement
*S* = 1.00	*w* = 1/[σ^2^(*F*_o_^2^) + (0.045*P*)^2^ + 0.7224*P*] where *P* = (*F*_o_^2^ + 2*F*_c_^2^)/3
2438 reflections	(Δ/σ)_max_ = 0.001
186 parameters	Δρ_max_ = 0.22 e Å^−^^3^
0 restraints	Δρ_min_ = −0.16 e Å^−^^3^

### Special details

**Table:**

Geometry. All e.s.d.'s (except the e.s.d. in the dihedral angle between two l.s. planes) are estimated using the full covariance matrix. The cell e.s.d.'s are taken into account individually in the estimation of e.s.d.'s in distances, angles and torsion angles; correlations between e.s.d.'s in cell parameters are only used when they are defined by crystal symmetry. An approximate (isotropic) treatment of cell e.s.d.'s is used for estimating e.s.d.'s involving l.s. planes.
Refinement. Refinement of *F*^2^ against ALL reflections. The weighted *R*-factor *wR* and goodness of fit *S* are based on *F*^2^, conventional *R*-factors *R* are based on *F*, with *F* set to zero for negative *F*^2^. The threshold expression of *F*^2^ > σ(*F*^2^) is used only for calculating *R*-factors(gt) *etc*. and is not relevant to the choice of reflections for refinement. *R*-factors based on *F*^2^ are statistically about twice as large as those based on *F*, and *R*- factors based on ALL data will be even larger.

### Fractional atomic coordinates and isotropic or equivalent isotropic displacement parameters (Å^2^)

**Table:**

	*x*	*y*	*z*	*U*_iso_*/*U*_eq_	
C1	0.2903 (2)	0.3204 (7)	0.3530 (6)	0.0620 (16)	
H1	0.2780	0.2284	0.3594	0.074*	
C2	0.2697 (2)	0.4365 (8)	0.4029 (7)	0.0732 (19)	
H2	0.2441	0.4229	0.4423	0.088*	
C3	0.2872 (2)	0.5721 (8)	0.3938 (7)	0.077 (2)	
H3	0.2740	0.6518	0.4279	0.092*	
C4	0.3246 (2)	0.5889 (7)	0.3340 (7)	0.0685 (17)	
H4	0.3367	0.6806	0.3257	0.082*	
C5	0.34421 (19)	0.4681 (6)	0.2858 (5)	0.0483 (13)	
C6	0.3844 (2)	0.4772 (6)	0.2209 (6)	0.0506 (13)	
C7	0.4082 (3)	0.5950 (7)	0.1822 (8)	0.079 (2)	
H7	0.4024	0.6926	0.1940	0.095*	
C8	0.4416 (3)	0.5372 (7)	0.1235 (8)	0.085 (2)	
H8	0.4632	0.5886	0.0868	0.102*	
C9	0.39499 (16)	0.0253 (5)	0.5065 (5)	0.0374 (11)	
C10	0.34569 (16)	−0.0362 (5)	0.4432 (5)	0.0367 (11)	
N1	0.32730 (15)	0.3343 (5)	0.2955 (5)	0.0472 (11)	
N2	0.40299 (15)	0.3533 (4)	0.1877 (5)	0.0470 (11)	
N3	0.43793 (17)	0.3939 (6)	0.1279 (5)	0.0628 (13)	
H3A	0.4556	0.3346	0.0965	0.075*	
Mn1	0.36805 (2)	0.14892 (8)	0.22637 (7)	0.0395 (3)	
O1	0.41292 (12)	0.1050 (4)	0.4304 (4)	0.0476 (9)	
O2	0.32916 (13)	−0.0076 (4)	0.3189 (4)	0.0514 (9)	
O3	0.41261 (12)	−0.0083 (4)	0.6295 (3)	0.0477 (9)	
O4	0.32667 (11)	−0.1102 (4)	0.5215 (3)	0.0433 (8)	
O1W	0.49441 (17)	0.2150 (6)	0.0153 (6)	0.0849 (14)	
O2W	0.5000	0.8860 (7)	0.2500	0.0754 (19)	
H1W	0.5197 (14)	0.181 (7)	0.056 (5)	0.080*	
H2W	0.485 (2)	0.183 (7)	−0.063 (4)	0.080*	
H3W	0.4792 (17)	0.944 (6)	0.222 (7)	0.080*	

### Atomic displacement parameters (Å^2^)

**Table:**

	*U*^11^	*U*^22^	*U*^33^	*U*^12^	*U*^13^	*U*^23^
C1	0.055 (3)	0.068 (4)	0.070 (4)	0.001 (3)	0.027 (3)	−0.007 (3)
C2	0.054 (4)	0.092 (5)	0.080 (5)	0.015 (4)	0.028 (3)	−0.012 (4)
C3	0.074 (4)	0.079 (5)	0.078 (5)	0.028 (4)	0.019 (4)	−0.021 (4)
C4	0.079 (4)	0.049 (3)	0.076 (4)	0.008 (3)	0.016 (4)	−0.018 (3)
C5	0.054 (3)	0.043 (3)	0.046 (3)	0.004 (2)	0.007 (2)	−0.007 (2)
C6	0.057 (3)	0.040 (3)	0.055 (3)	−0.003 (2)	0.012 (3)	−0.002 (2)
C7	0.101 (5)	0.041 (3)	0.104 (6)	−0.009 (3)	0.039 (5)	0.007 (3)
C8	0.091 (5)	0.065 (4)	0.112 (6)	−0.022 (4)	0.047 (5)	0.013 (4)
C9	0.042 (3)	0.035 (3)	0.037 (3)	−0.002 (2)	0.013 (2)	−0.003 (2)
C10	0.043 (3)	0.032 (2)	0.038 (3)	−0.003 (2)	0.013 (2)	−0.004 (2)
N1	0.048 (2)	0.046 (3)	0.051 (3)	0.0022 (19)	0.017 (2)	−0.0065 (19)
N2	0.051 (3)	0.041 (2)	0.053 (3)	−0.0033 (19)	0.021 (2)	0.0016 (19)
N3	0.061 (3)	0.063 (3)	0.074 (3)	−0.006 (2)	0.035 (3)	0.008 (3)
Mn1	0.0490 (5)	0.0355 (5)	0.0370 (5)	0.0002 (3)	0.0160 (3)	−0.0001 (3)
O1	0.046 (2)	0.055 (2)	0.043 (2)	−0.0140 (16)	0.0116 (16)	0.0073 (16)
O2	0.056 (2)	0.059 (2)	0.037 (2)	−0.0187 (18)	0.0051 (16)	0.0048 (16)
O3	0.046 (2)	0.057 (2)	0.039 (2)	−0.0124 (16)	0.0069 (16)	0.0062 (16)
O4	0.0449 (19)	0.0467 (19)	0.0401 (19)	−0.0105 (15)	0.0132 (16)	0.0017 (15)
O1W	0.060 (3)	0.103 (4)	0.097 (4)	0.016 (3)	0.027 (3)	−0.002 (3)
O2W	0.047 (4)	0.069 (4)	0.102 (5)	0.000	−0.002 (4)	0.000

### Geometric parameters (Å, °)

**Table:**

C1—N1	1.342 (7)	C9—O3	1.250 (6)
C1—C2	1.376 (8)	C9—C10	1.557 (7)
C1—H1	0.9300	C10—O2	1.245 (6)
C2—C3	1.365 (10)	C10—O4	1.253 (5)
C2—H2	0.9300	N2—N3	1.348 (6)
C3—C4	1.367 (9)	N3—H3A	0.8600
C3—H3	0.9300	Mn1—N1	2.280 (4)
C4—C5	1.388 (8)	Mn1—N2	2.223 (4)
C4—H4	0.9300	Mn1—O4^i^	2.150 (3)
C5—N1	1.344 (6)	Mn1—O2	2.168 (3)
C5—C6	1.468 (8)	Mn1—O1	2.191 (4)
C6—N2	1.339 (7)	Mn1—O3^i^	2.208 (3)
C6—C7	1.392 (8)	O3—Mn1^ii^	2.208 (3)
C7—C8	1.357 (10)	O4—Mn1^ii^	2.150 (3)
C7—H7	0.9300	O1W—H1W	0.83 (5)
C8—N3	1.329 (8)	O1W—H2W	0.82 (4)
C8—H8	0.9300	O2W—H3W	0.82 (5)
C9—O1	1.250 (6)		
			
N1—C1—C2	122.7 (6)	C1—N1—C5	117.8 (5)
N1—C1—H1	118.7	C1—N1—Mn1	125.8 (4)
C2—C1—H1	118.7	C5—N1—Mn1	116.2 (3)
C3—C2—C1	119.2 (6)	C6—N2—N3	105.2 (4)
C3—C2—H2	120.4	C6—N2—Mn1	117.0 (3)
C1—C2—H2	120.4	N3—N2—Mn1	137.6 (4)
C2—C3—C4	119.0 (6)	C8—N3—N2	111.5 (5)
C2—C3—H3	120.5	C8—N3—H3A	124.2
C4—C3—H3	120.5	N2—N3—H3A	124.2
C3—C4—C5	119.5 (6)	O4^i^—Mn1—O2	92.44 (13)
C3—C4—H4	120.3	O4^i^—Mn1—O1	159.58 (14)
C5—C4—H4	120.3	O2—Mn1—O1	75.93 (13)
N1—C5—C4	121.7 (5)	O4^i^—Mn1—O3^i^	76.27 (12)
N1—C5—C6	115.5 (4)	O2—Mn1—O3^i^	102.10 (16)
C4—C5—C6	122.8 (5)	O1—Mn1—O3^i^	89.63 (13)
N2—C6—C7	110.1 (5)	O4^i^—Mn1—N2	99.67 (15)
N2—C6—C5	118.1 (4)	O2—Mn1—N2	161.17 (16)
C7—C6—C5	131.8 (5)	O1—Mn1—N2	96.12 (15)
C8—C7—C6	105.4 (6)	O3^i^—Mn1—N2	94.79 (14)
C8—C7—H7	127.3	O4^i^—Mn1—N1	100.36 (14)
C6—C7—H7	127.3	O2—Mn1—N1	90.74 (16)
N3—C8—C7	107.8 (6)	O1—Mn1—N1	96.58 (15)
N3—C8—H8	126.1	O3^i^—Mn1—N1	166.79 (15)
C7—C8—H8	126.1	N2—Mn1—N1	73.01 (16)
O1—C9—O3	126.2 (4)	C9—O1—Mn1	114.4 (3)
O1—C9—C10	117.0 (4)	C10—O2—Mn1	115.4 (3)
O3—C9—C10	116.8 (4)	C9—O3—Mn1^ii^	114.0 (3)
O2—C10—O4	126.4 (4)	C10—O4—Mn1^ii^	115.7 (3)
O2—C10—C9	116.5 (4)	H1W—O1W—H2W	114 (4)
O4—C10—C9	117.1 (4)		

Symmetry codes: (i) *x*, −*y*, *z*−1/2; (ii) *x*, −*y*, *z*+1/2.

### Hydrogen-bond geometry (Å, °)

**Table:**

*D*—H···*A*	*D*—H	H···*A*	*D*···*A*	*D*—H···*A*
N3—H3A···O1W	0.86	1.89	2.748 (7)	175
O1W—H1W···O1^iii^	0.83 (5)	2.08 (4)	2.851 (6)	155 (6)
O1W—H2W···O2W^iv^	0.82 (4)	2.10 (5)	2.819 (6)	148 (6)
O2W—H3W···O3^v^	0.82 (5)	2.06 (4)	2.823 (4)	156 (6)

Symmetry codes: (iii) −*x*+1, *y*, −*z*+1/2; (iv) −*x*+1, −*y*+1, −*z*; (v) *x*, −*y*+1, *z*−1/2.
